# Enabling Targeted Drug Delivery for Treatment of Ulcerative Colitis with Mucosal‐Adhesive Photoreactive Hydrogel

**DOI:** 10.1002/advs.202404836

**Published:** 2025-02-03

**Authors:** Wen Wu, Jian Zhang, Xiao Qu, Ting Chen, Jinming Li, Yongzhi Yang, Lifeng Chen, Alex Hoover, Fanying Guo, Cheng Kong, Bingkun Bao, Qiuning Lin, Mengxin Zhou, Linyong Zhu, Xiaoyang Wu, Yanlei Ma

**Affiliations:** ^1^ Department of Colorectal Surgery Fudan University Shanghai Cancer Center Department of Oncology Shanghai Medical College Fudan University No. 270 Dongan Road Shanghai 200032 China; ^2^ Ben May Department for Cancer Research University of Chicago GCIS W408B, 929 E 57th Street Chicago IL 60637 USA; ^3^ Department of Endoscopy Fudan University Shanghai Cancer Center No. 270 Dongan Road Shanghai 200032 China; ^4^ School of Biomedical Engineering Shanghai Jiao Tong University No. 800 Dongchuan Road Shanghai 200240 China; ^5^ School of Chemistry and Molecular Engineering East China University of Science and Technology No.130 Meilong Road Shanghai 200237 China

**Keywords:** endoscopic drug delivery, photoreactive hydrogel, ulcerative colitis

## Abstract

Ulcerative colitis (UC) is a chronic inflammatory bowel disease. UC treatments are limited by significant adverse effects associated with non‐specific drug delivery, such as systematic inhibition of the host immune system. Endoscopic delivery of a synthetic hydrogel material with biocompatible gelation that can efficiently cover irregular tissue surfaces provides an effective approach for targeted drug delivery at the gastrointestinal (GI) tract. An ideal integration of synthetic material with intestinal epithelium entails an integrated and preferable chemically bonded interface between the hydrogel and mucosal surface. In this study, a photo‐triggered coupling reaction is leveraged as the crosslinking platform to develop a mucosal‐adhesive hydrogel, which is compatible with endoscope‐directed drug delivery for UC treatment. The results demonstrated superior spatiotemporal specificity and drug pharmacokinetics with this delivery system in vivo. Delivery of different drugs with the hydrogel leads to greatly enhanced therapeutic efficacy and significantly reduced systemic drug exposure with rat colitis models. The study presents a strategy for targeted and persistent drug delivery for UC treatment.

## Graphical abstract

This study introduces a novel photo‐triggered coupling reaction to develop a mucosal‐adhesive hydrogel for endoscopic delivery in ulcerative colitis (UC) treatment. The hydrogel, with its biocompatible gelation and efficient coverage of irregular tissue surfaces, offers targeted drug delivery at the gastrointestinal tract, reducing systemic drug exposure and enhancing therapeutic efficacy. This innovative approach presents significant potential for improving UC treatment outcomes by minimizing adverse effects associated with non‐specific drug delivery methods.

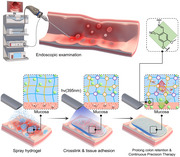

## 1 Introduction

Inflammatory bowel disease (IBD) describes chronic intestinal disorders, which include Crohn's disease (CD) and ulcerative colitis (UC).^[^ ^1^ ^]^ Whereas UC is limited to the colon, CD can affect any section of the intestine, usually in a noncontiguous manner. The pathophysiology of IBD, such as UC, involves complex genetic, environmental, microbial, and immune factors. Although increased knowledge of disease etiology has promoted the advance in drug development during the last decades with the approval of several novel biological and small‐molecule medicines, remission rates of UC patients given new therapeutic agents in clinical trials remain at a modest 20–30%.^[^ ^2^ ^]^ This therapeutic “ceiling” highlights the urgent need for alternative strategies to devise a more effective treatment for UC.

The therapeutic potential of conventional UC treatments is limited by significant adverse effects due to non‐specific drug delivery, such as systemic inhibition of the host immune system.^[^ ^2^ ^]^ The success of UC treatment is thus critically dependent upon the effective delivery of the drug to the target tissue, which can reduce the side effects while improving therapeutic efficacy. Endoscopy is a routine procedure for the diagnosis, management, and treatment of UC.^[^ ^3^ ^]^ As endoscopic technologies have progressed, novel tools such as endoscopic ultrasound and balloon‐assisted enteroscopy have been developed and widely used for the treatment of UC. Endoscopy can also serve as a potential drug delivery platform. Endoscopic injection of steroids and TNF‐α antibodies have been attempted. However, the clinical benefits of direct drug injection with endoscopes remain unclear.^[^ ^4^ ^]^

In the past decades, the increased availability of new biomaterials (including liposomes, nanoparticles, organic carriers, and hydrogels) has promoted the development of different drug carriers. Compared to conventional therapies, new drug delivery platforms have demonstrated the potential to overcome the challenges of different disease treatments by effectively changing drug biodistribution, pharmacokinetics, and systemic toxicity. However, despite the plethora of pre‐clinical studies, only a few targeted carriers have been approved for clinical use, such as Doxil and Abraxane for cancer therapy. Biocompatibility, manufacturing challenges, and the complexity of the tissue microenvironment have limited the clinical benefits of different drug delivery systems.^[^ ^5^ ^]^

In this study, we designed a synthetic hydrogel material with biocompatible gelation, which can efficiently adhere and cover irregular tissue surfaces in vivo, providing an effective way for endoscope‐guided drug delivery (Figure S1, Supporting Information). An ideal integration of synthetic material with intestinal epithelium entails an integrated and preferable chemically bonded interface between the hydrogel and mucosal surface. Although various macromolecules bearing dual‐reactivity for both biological surface and pre‐gelling polymers have been tested, they usually require multistep operation for gelling or harsh oxidation conditions that impair the biocompatibility of the material.^[^ ^6^ ^]^ To address these issues, we leverage the photo‐triggered transient‐radical and persistent‐radical coupling (PTPC) reaction^[^ ^7^ ^]^ to develop photo‐crosslinked hyaluronic acid hydrogels that consist of *o*‐nitrobenzyl (NB)‐grafted hyaluronic acid (HANB) and methacrylated hyaluronic acid (HAMA), for controlled drug release at GI tract. The photo‐crosslinked hyaluronic acid (HANB/HAMA) hydrogels are biocompatible and can achieve fast gelling in situ upon UV irradiation. Under irradiation, the nitroxides derived from *o*‐nitrobenzyl alcohol (NB) are uniquely persistent and oxygen insensitive, which exhibit low self‐reactivity and specifical reactivity with free radicals.^[^ ^7^ ^]^ Moreover, the aldehyde groups generated from *o*‐ nitrobenzyl (NB) can rapidly react with the amino groups on the mucosal surface of intestinal tissue, ensuring strong tissue adhesion through covalently anchoring in vivo. The highly efficient PTPC reaction provides a more robust hydrogel than photo‐initiated radical polymerization;^[^ ^7^, ^8^ ^]^ and the responsive generation of reactive aldehyde groups facilitates the spatiotemporally controlled adhesion in situ rather than partially oxidized polysaccharides‐based hydrogel.^[^ ^6^, ^9^ ^]^ Drug‐laden HANB/HAMA hydrogels can be readily deployed by conventional endoscopic systems through pressurized spraying and UV‐induced adhesion to inflamed colonic epithelium. With rat colitis models, our results demonstrated that the drug delivery platform can significantly enhance the therapeutic efficacy of different UC drugs, including budesonide and mesalazine. Endoscope‐guided delivery can alleviate the inflammatory response, promote intestinal barrier repair, reduce tissue fibrosis, and balance intestinal flora while reducing systemic side effects of the drugs in vivo. Our study presents a targeted drug delivery platform for UC treatment.

## 2 Results

### 2.1 Formulation and Characterization of a Biodegradable Hydrogel for Intestinal Drug Delivery

**Figure 1:**
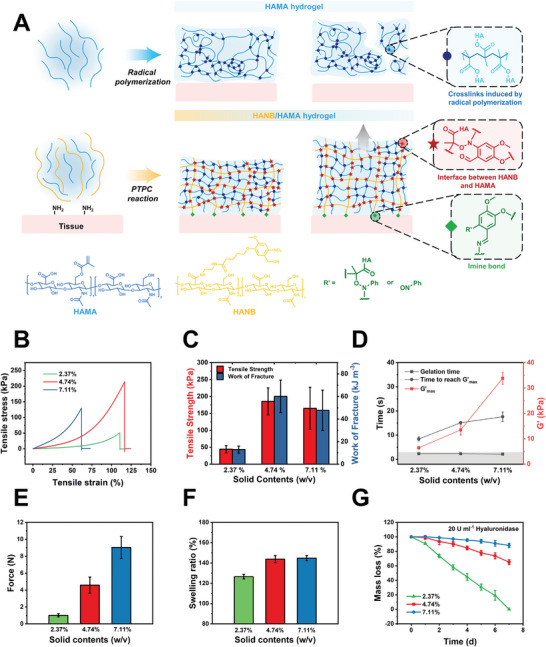
The design and characterization of HANB/HAMA hydrogels. A) Schematic illustration of mechanisms for HANB/HAMA hydrogel construction. B) Representative tensile stress‐strain curves of HANB/HAMA hydrogels with solid contents of 2.37, 4.74, and 7.11%. C) Tensile strength and work of fracture of HANB/HAMA hydrogels with solid contents of 2.37, 4.74, and 7.11%. D) Rheology analysis of HANB/HAMA hydrogels with solid contents of 2.37, 4.74, and 7.11%. E) Injection force of HANB/HAMA hydrogels with solid contents of 2.37, 4.74, and 7.11%. F) The swelling ratio of HANB/HAMA hydrogels with solid contents of 2.37, 4.74, and 7.11% in PBS (pH = 7.4). G) Degradation profiles of HANB/HAMA hydrogels with solid contents of 2.37, 4.74, and 7.11% in hyaluronidase‐supplemented PBS (20 U ml^−1^, pH = 7.4). Data are presented as mean ± s.d. (*n* = 3).

To enhance tissue adhesion, we have designed a photo‐triggered transient‐radical and persistent‐radical coupling reaction (abbreviated as PTPC reaction) to fabricate biomimetic interfacial‐bonding nanocomposite hydrogels,^[^ ^7^ ^]^ which can overcome the shortage of traditional hydrogels formed by photo‐initiated radical polymerization, such as the poor mechanical properties and the lack of tissue adhesion. However, swelling of the hydrogel carrier may lead to intestinal obstruction. In consideration of the swelling profile of polyethylene glycol (PEG),^[^ ^10^ ^]^ we synthesized the *o*‐nitrobenzyl (NB)‐grafted hyaluronic acid (HANB) to replace NB‐terminated tetra‐armed PEG (PEGNB) and prepared HANB/HAMA hydrogels (**Figure** **1**A).^[^ ^7^ ^]^ To construct the HANB/HAMA hydrogel, HANB and methacrylated hyaluronic acid (HAMA) were first synthesized (Figure S2, Supporting Information) as described in our previous report.^[^ ^11^ ^]^ We chose HA, a glycosaminoglycan (GAG) in the extracellular matrix (ECM),^[^ ^12^ ^]^ as the backbone of the hydrogel because of its wide use as biocompatible and degradable biomaterials,^[^ ^13^ ^]^ its characteristics of tissue repair and regeneration, and its anti‐inflammatory effect by promoting M1 to M2 polarization.^[^ ^14^ ^]^ The precursor solution of the HANB/HAMA network includes HANB (MW = 340 kDa) with 4% substituted degree (s.d.), HAMA (Mw = 44 kDa, 69% s.d.), and a lithium phenyl‐2,4,6‐trimethylbenzoylphosphinate (LAP) photo‐initiator, which can improve the strength of hydrogels and provide the tissue adhesive properties.

The mechanical profiles of HANB/HAMA hydrogels with different formulations [2.37% (w/v), HANB/HAMA/LAP = 2:0.25:0.12; 4.74% (w/v), HANB/HAMA/LAP = 4:0.5:0.24; 7.11% (w/v), HANB/HAMA/LAP = 6:0.75:0.36) were different. Compared with four kinds of HAMA hydrogels [HAMA*‐ 1*, Mw = 44 kDa, 69% s.d., 0.5% (w/v); HAMA*‐2*, Mw = 44 kDa, 69% s.d., 4% (w/v); HAMA*‐3*, Mw = 340 kDa, 10% s.d., 4% (w/v); HAMA*‐4*, Mw = 340 kDa, 20% s.d., 4% (w/v)], all HANB/HAMA hydrogels display good mechanical performance with excellent elasticity and strength (Video S1, Table S1, Supporting Information), suggesting the introduction of HANB enhanced the mechanical properties of HA hydrogels via efficient PTPC reaction. With the elevation of solid contents, the HANB/HAMA hydrogels demonstrate higher compression strength due to increased crosslinking density and polymer entanglement (Figure S3A, Supporting Information). Repeated compression tests at strains of 10, 20, and 30% revealed no obvious reduction in the mechanical properties of the HANB/HAMA gels over the compression cycles (Figure S3B, Supporting Information). Notably, as shown in Figures 1B,C, the HANB/HAMA hydrogel with a solid content of 4.74% achieves the longest elongation (116.25 ± 0.43%), the highest tensile strength (200.675 ± 13.025 kPa), and the highest work of fracture (60.094 ± 14.458 kJ m^−3^). All HANB/HAMA hydrogels gelled rapidly within 3 s through the PTPC reaction (Figure 1D; Figure S4A, Supporting Information). Before irradiation, all precursors demonstrated liquid properties (G″ > G′), and after irradiation, all G′ values were stable and greater than G″ during the elevation of stress, indicating complete gelation (Figure S4B, Supporting Information). Notably, under high stress, the precursors with higher solid contents (4.74 and 7.11%) show a thinning characteristic, which might be beneficial for injection. However, in contrast to the highest solid content, the 2.37 and 4.74% solid content endowed the hydrogels with injectability at 37 °C, which is critical for endoscopic delivery (Figure 1E). We chose the hydrogel with 4.74% solid content for drug delivery in vivo.

Swelling and degradation are crucial properties in determining the physiological fates of implanted hydrogels. The swelling ratio of HANB/HAMA hydrogels with the solid contents of 2.37, 4.74, and 7.11% in PBS (pH = 7.4) is 126.51 ± 2.19%, 143.83 ± 3.53%, and 144.78 ± 2.59%, respectively (Figure 1F), which is significantly lower compared with the surgical sealant COSEAL (swelling ratio up to 400%, Table S2, Supporting Information),^[^ ^15^ ^]^ lowering the risk of bowel obstruction. The swelling ratio of the HANB/HAMA hydrogel is comparable at different pH for intracolonic application (Figures S5A,B, Supporting Information). After reaching complete swelling equilibrium, HANB/HAMA hydrogel samples were immersed in PBS supplemented with hyaluronidase (from 20 to 100 U mL^−1^) to test biodegradation in vivo (Figure 1G; Figure S5C, Supporting Information). As the solid contents decreased or concentrations of hyaluronidase increased, the HANB/HAMA hydrogel demonstrated an accelerated degradation rate. HANB/HAMA hydrogel also demonstrates good biocompatibility. Co‐culture experiments indicate no significant cytotoxicity to either NC460 or Caco‐2 cells in vivo, as determined by both cell viability test and cell live‐dead staining (Figure S6, Supporting Information). Moreover, the hydrogels were implanted subcutaneously and intraperitoneally in rats to evaluate their biodegradation and safety in vivo. Over a period of 42 days, the rats exhibited normal behavior, indicating no adverse effects from the implantation. The mass degradation rates of HANB/HAMA and control hydrogels in subcutaneous tissue were 69.5 ± 11.7% and 65.8 ± 12.3%, respectively. In contrast, the degradation rates of the two hydrogels in the abdominal cavity were 38.9 ± 7.7% and 29.2 ± 4.9%, respectively. These findings suggest that both hydrogels undergo gradual biodegradation over time, as shown in (Figure S7, Supporting Information).

### 2.2 HANB/HAMA Hydrogel has Superior Tissue Adhesive Capability

**Figure 2:**
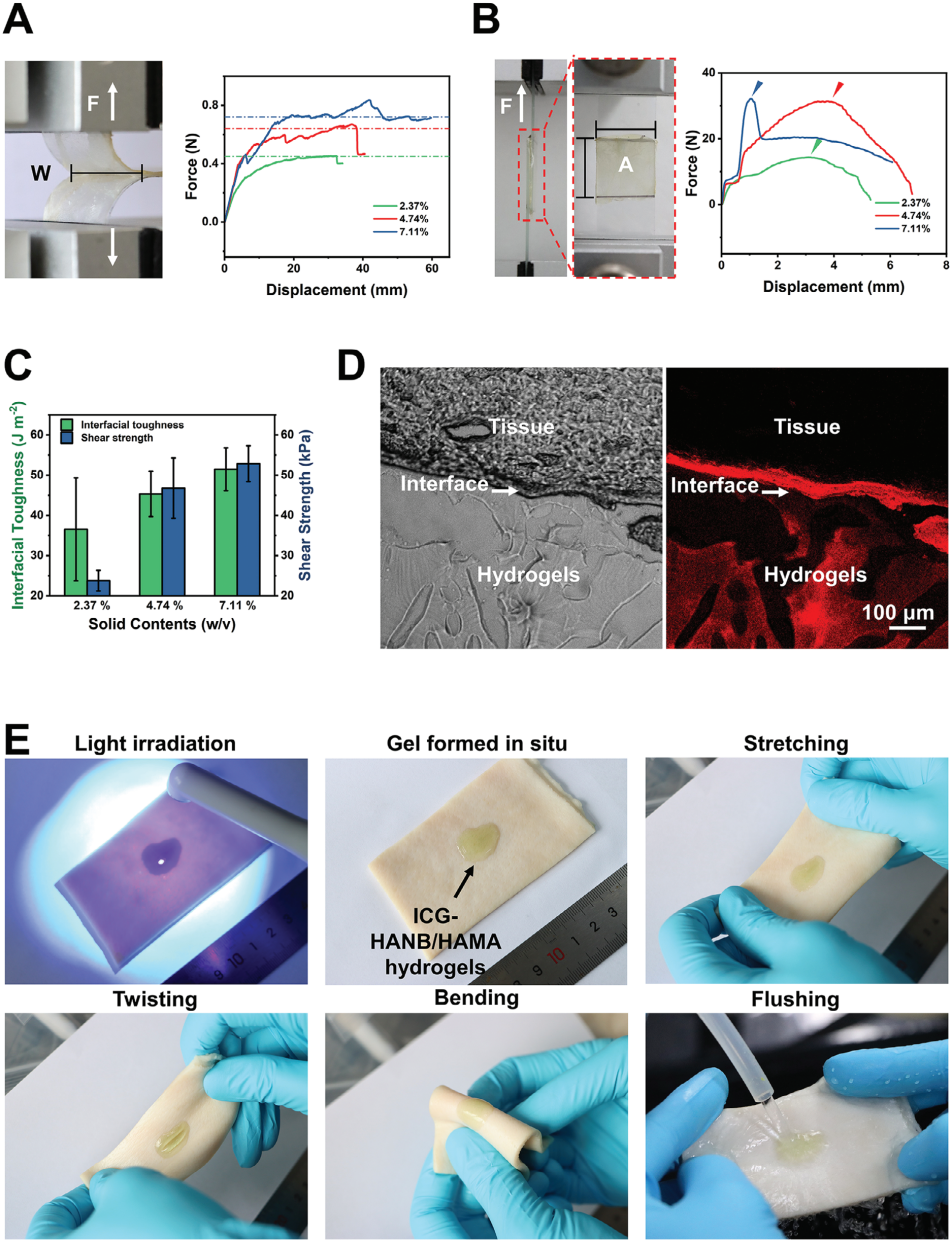
Tissue adhesion of HANB/HAMA hydrogels. A) Standard 180‐degree peeling tests to determine the interfacial toughness of HANB/HAMA hydrogels with solid contents of 2.37, 4.74, and 7.11%. B) Standard lap shear tests to determine the shear strength of HANB/HAMA hydrogels with solid contents of 2.37, 4.74, and 7.11%. C) Interfacial toughness and shear strength of HANB/HAMA hydrogels with solid contents of 2.37, 4.74, and 7.11%. D) The Bright field and fluorescence image of hydrogel–porcine skin constructs. E) Photographs of the tissue adhesion of HANB/HAMA hydrogels on fresh porcine skin wetted by PBS. Data are presented as mean ± s.d. (*n* = 3).

To determine tissue adhesion, based on the ASTM F2256 and ASTM F2255‐05, standard 180‐degree peel testing, and lap shear testing were performed to determine the tissue‐adhesive properties of HANB/HAMA hydrogels. Those tests were carried out by using hog casing composites as substrate (as shown in the insets of **Figures** **2**A,B). With the elevation of the solid content, the hydrogels exhibited higher interfacial toughness and shear strength (Figure 2C). The HANB/HAMA hydrogel with a solid content of 7.11% demonstrates an interfacial toughness of 51.47 ± 5.33 J m^−2^ and a shear strength of 52.85 ± 4.43 kPa. Whereas the HANB/HAMA hydrogel with a solid content of 4.74% demonstrates a comparable tissue adhesion property in both criteria (an interfacial toughness of 45.33 ± 5.62 J m^−2^ and a shear strength of 46.77 ± 7.49 kPa). Both criteria were significantly higher than that of HAMA hydrogels which have no aldehyde groups derived from NB groups (Table S1, Supporting Information). Considering the tissue adaptiveness, injectability, and adhesive capability, we selected the HANB/HAMA hydrogel with a solid content of 4.74% as the optimal hydrogel to inject through catheters, deliver drugs, adhere to the mucosa, and withstand the dynamic environment.

To further evaluate the tissue adhesive property of the material, we deposited Indocyanine Green (ICG)‐labeled HANB/HAMA hydrogel precursors onto wet porcine skin, which gelled in situ under UV irradiation (395 nm LED, 50 mW cm^−2^). To further inspect the robust tissue adhesion of HANB/HAMA hydrogels on the porcine skin, we utilize fluorescence microscopy to examine the integrated interface between hydrogels and the tissue surface. The fluorescence image unveiled a compact interaction between the porcine skin and HANB/HAMA hydrogels (Figure 2D). Therefore, the HANB/HAMA hydrogels tightly adhered to the wet tissue surface despite repeated stretching, twisting, bending, and flushing with water (Figure 2E and Video S2, Supporting Information).

**Figure 3:**
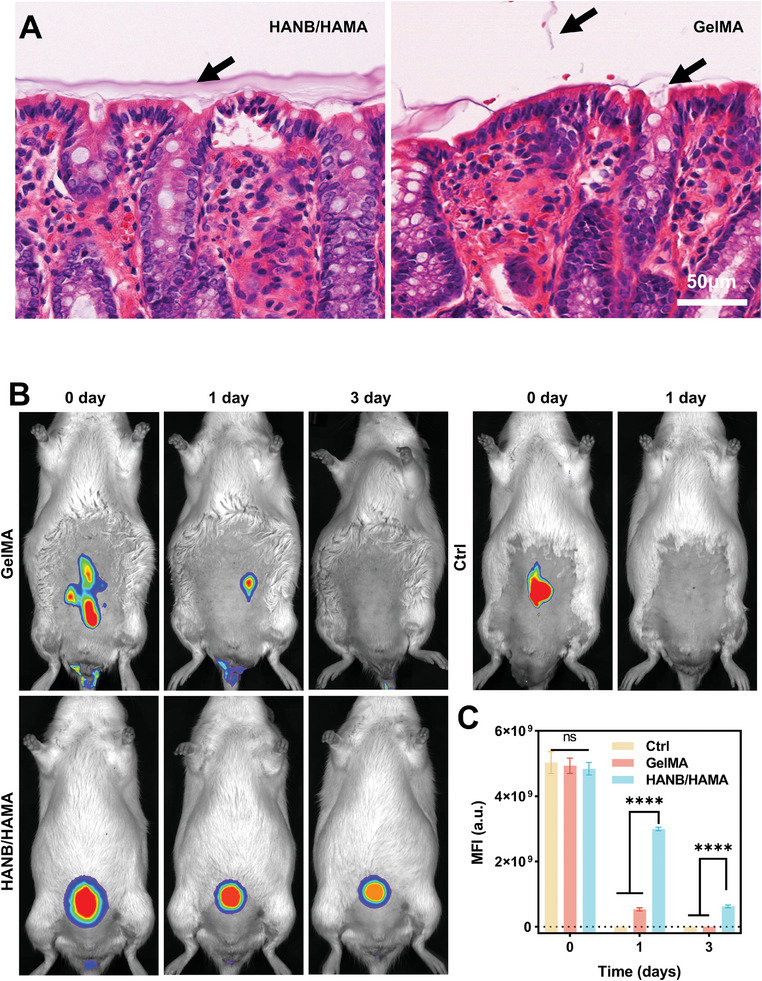
The HANB/HAMA hydrogel displayed superior inflamed colonic adhesion and long retention. A) Representative HE staining of HANB/HAMA (left, 3 days) and GelMA (right, 1 day) Hydrogels adhered to colon mucosa after endoscopic injection. The dark arrow marked the spot where the HANB/HAMA or GelMA hydrogels adhered to the mucosa, respectively (scale bar: 50 µm). B) Fluorescence of ICG‐labeled different hydrogels was detected to assess colonic retention time by IVIS imaging system in DSS‐induced rats. C) The corresponding mean fluorescence intensity (MFI) of the ICG‐labeled hydrogels at different time points. All data are presented as mean ± s.d. (*n* = 6). Statistical analysis was performed using two‐way ANOVA with Tukey's multiple comparison test. ^*^ *P* < 0.05, ^**^ *P* < 0.01, ^***^ *P* < 0.001, ^****^ *P* < 0.0001.

To examine the adhesive capability to the intestinal mucosal surface, we delivered the HANB/HAMA precursor to the rat colon and induced hydrogel formation in situ by endoscope‐guided UV irradiation (Video S3, Supporting Information). We used a small animal endoscope from KARL STORZ company with working channels for the injection of hydrogels. The hydrogel was injected under pressure from the syringe with a flexible metal tube through the channel (5Fr, 1.7 mm) of endoscopy. The tube's outer diameter is 0.6 mm, its inner diameter is 0.3 mm and the length of the needle is 500 mm (Video S4, Supporting Information). As soon as the drug‐loaded HANB/HAMA precursor solution was injected at the inflamed site, the 395 nm UV light (50 mW cm^−2^) was turned on for 10 s to form the HANB/HAMA hydrogel in situ. The light irradiation was generated by a UV light machine (OUTPUT 800 mV, Model No. TCS‐1.5‐AA‐FE1S) through a dedicated UV optical fiber (diameter 1.2 mm). Endoscopic images and histology analysis indicate that HANB/HAMA hydrogel can firmly adhere to the mucosa after delivery (**Figure** **3**A; Figure S8, Supporting Information). By contrast, the control hydrogel methacrylate gelatin [GelMA, 20% (w/v), 30% s.d., with 0.2% (w/v) LAP] showed significant detachment and limited adhesion 1‐day post‐delivery (Figure 3A). To monitor hydrogel retention in the colon, we delivered fluorescent ICG‐HANB/HAMA hydrogel to the rat colon with an endoscope. HANB/HAMA hydrogel exhibits markedly enhanced retention in vivo in comparison with GelMA or free ICG solution (Figure 3B and quantification in Figure 3C).

### 2.3 HANB/HAMA Hydrogel can Improve Drug Pharmacokinetics (PK) and Biodistribution for UC Treatment

The drug release properties of hydrogel were dependent on the crosslinking density, degradation, and microenvironment. Both budesonide and mesalazine are common drugs used for UC treatments. When tested in vivo, HANB/HAMA hydrogel exhibits slow and persistent release of budesonide, whereas mesalazine released significantly faster from the hydrogel (Figures S9A,B, Supporting Information), likely due to more extensive hydrogen bond formation and crosslinking between budesonide with the hydrogel.

**Figure 4:**
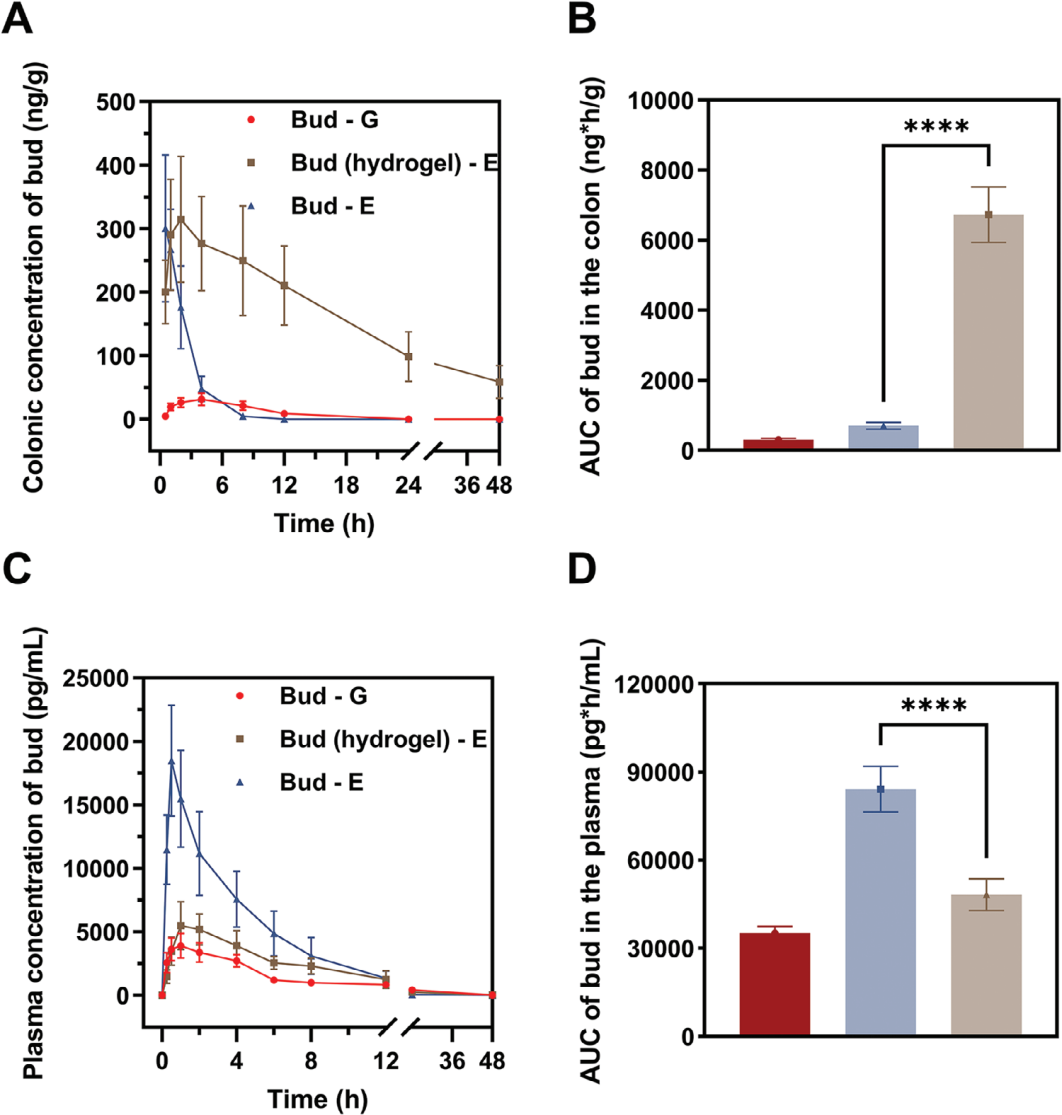
The Bud‐loaded HANB/HAMA displayed superior pharmacokinetics and higher bud concentrations of inflamed sites. A) Quantitative analysis of Bud distribution in the colon. B) Cumulative amount of Bud in the colon by calculating the area under the curve (AUC). C,D) The drug distribution (C) and cumulative amount of Bud (D) in the plasma of colitis mice. All data are presented as mean ± s.d. (*n* = 6). Statistical analysis was performed using an unpaired two‐tailed Student t‐test. ^*^ *P* < 0.05, ^**^ *P* < 0.01, ^***^ *P* < 0.001, ^****^ *P* < 0.0001, Bud (hydrogel) – E versus Bud – E.

To determine the effect of HANB/HAMA on drug PK in vivo, we delivered budesonide to rats via endoscope‐assisted delivery with hydrogel, conventional oral gavage, or drug solution enema. We then analyzed the plasma or colonic concentration of budesonide by mass spectrometry. Although both enema and hydrogel‐mediated delivery reach similar maximum concentrations of budesonide (C_max_) in the colon, hydrogel delivery leads to much more durable drug release in vivo (**Figure** **4**A). The cumulative drug exposure from hydrogel‐mediated delivery is 9.59 times higher than the drug solution enema (Figure 4B). By contrast, delivery with the HANB/HAMA hydrogel can significantly reduce C_max_ and cumulative drug exposure in plasma, compared with enema‐mediated delivery. (Figures 4C,D). Hydrogel delivery reduces plasma C_max_ by 70.3% and overall drug release by 42.7%. When we used DDI (drug delivery index)^[^ ^16^ ^]^ to evaluate the colonic relative targeting efficiency of budesonide, HANB/HAMA‐mediated delivery can achieve 16.06, compared with enema. Taken together, our results provide compelling evidence that HANB/HAMA hydrogels can effectively enhance targeted drug release and increase the colonic bioavailability of budesonide for UC treatment.

### 2.4 Drug Delivery with HANB/HAMA Hydrogel Enhances Therapeutic Efficacy for UC

**Figure 5:**
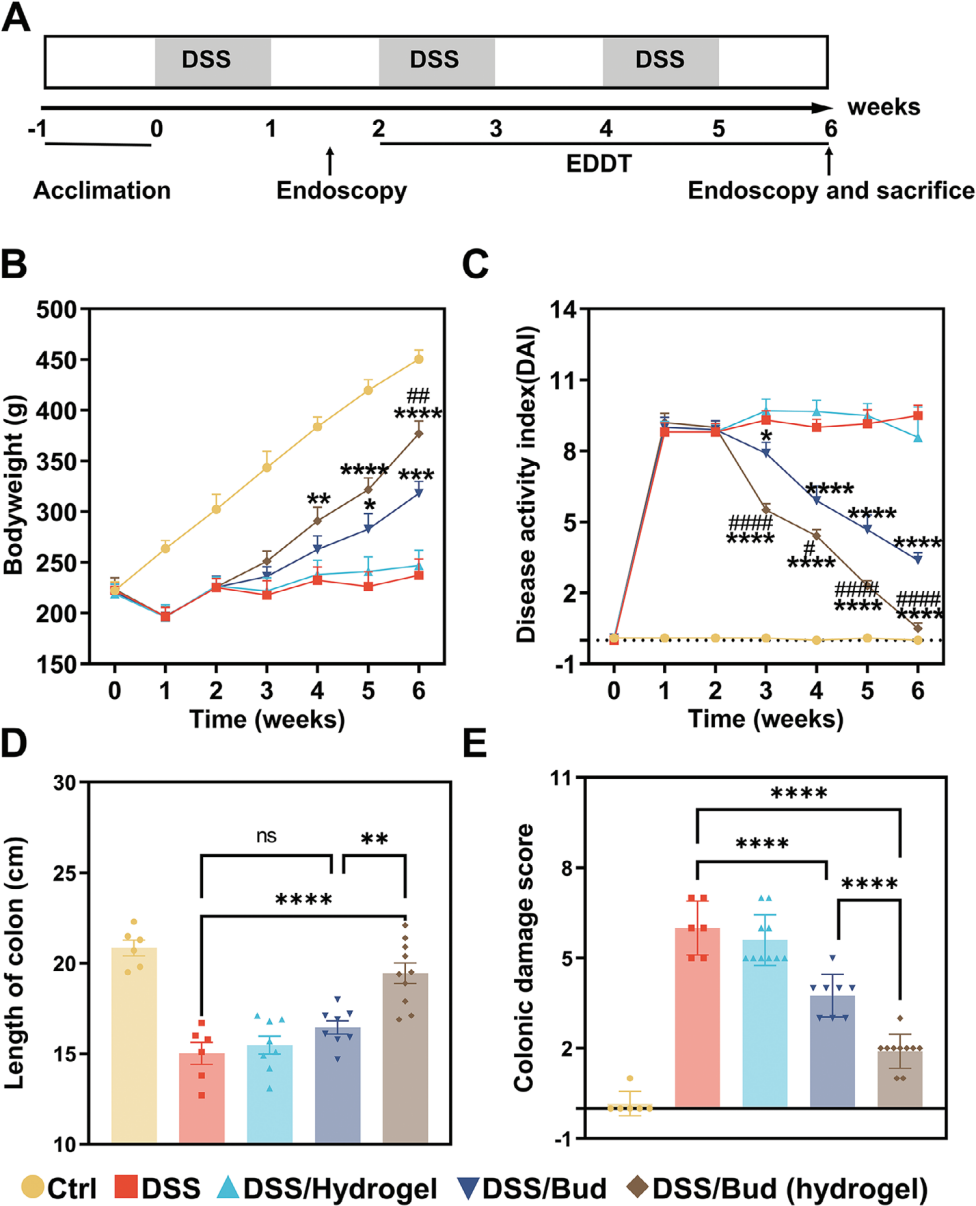
Bud‐loaded HANB/HAMA hydrogel exerts stronger efficacy in chronic colitis rats. A) Schematic diagram of DSS‐induced chronic colitis. EDDT, endoscopic drug delivery therapy. B,C) Bodyweight (B) and disease activity index (DAI) changes (C) in each group for 6 weeks. D) Quantitative colon length of each group at the end of the experiment. E) Colonic damage scores of each group at the end of the experiment. Data are presented as the mean ± s.e.m. (*n* = 10). Statistical analysis was performed using two‐way or one‐way ANOVA with Tukey's multiple comparison test. ^*^ *P* < 0.05, ^**^ *P* < 0.01, ^***^ *P* < 0.001, ^****^ *P* < 0.0001, DSS/Bud (hydrogel) or DSS/Bud versus DSS. ^#^ *P* < 0.05, ^##^ *P* < 0.01, ^###^ *P* < 0.001, ^####^ *P* < 0.0001, DSS/Bud (hydrogel) versus DSS/Bud.

To examine the therapeutic potential of HANB/HAMA‐mediated drug delivery, we used DSS induced chronic enteritis model in rats (**Figure** **5**A). Delivery of budesonide through endoscopy with the hydrogel (at a dose of 0.5 mg kg^−1^ d^−1^ for every 3 days) leads to significantly improved body weight gain and reduced disease activity index (DAI), compared with the control group or rats treated with budesonide through enema (Figures 5B,C). When evaluated by endoscopy, the colon of control animals has well‐formed vascular patterns and smooth surfaces without signs of colitis, as indicated by diarrhea, bleeding, or the appearance of fibrosis. However, the colon of rats in the DSS treatment group exhibits strong damage, including colon roughness, bleeding, fibrosis, and stricture (Figure S10A, Supporting Information). Delivery of budesonide through the hydrogel significantly reduces the symptoms and leads to quantitative improvement in the Mayo endoscopic score (Figures S10A,B, Supporting Information). Consistently, we also observed increased colon length and decreased colonic damage score (quantification of severity of inflammation, depth of inflammation, and extent of gland damage) when hydrogel‐mediated drug delivery is used (Figures 5D,E). Delivery of mesalazine with HANB/HAMA hydrogel can achieve a similar improvement in therapeutic efficacy with the rat colitis model (Figure S11, Supporting Information).

Biosafety of hydrogel materials is critical for future clinical translation. Animals treated with drug‐laden hydrogel exhibited no abnormal signs of intestinal obstruction and necrosis, and there were no significant differences in liver function (Figures S12A–C, Supporting Information) and kidney function (Figures S12D–F, Supporting Information). Histology of the major organs, including the heart, lung, liver, spleen, and kidneys in treated animals are normal, confirming the biocompatibility of the hydrogel in vivo (Figure S13, Supporting Information).

### 2.5 Endoscope‐Guided Drug Delivery Alleviates Inflammation and Tissue Fibrosis in the Colitis Model

**Figure 6:**
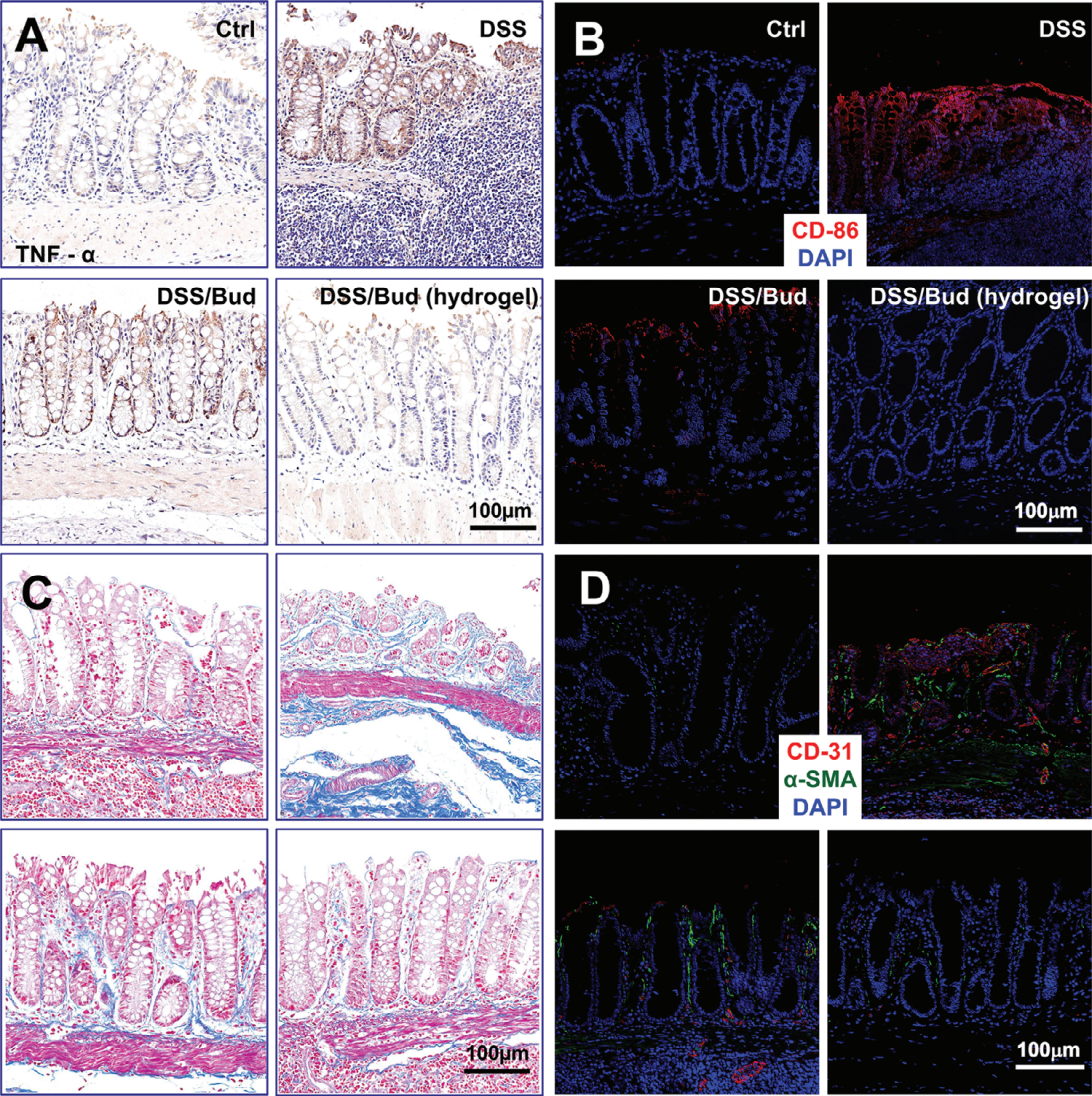
Inhibited inflammation and immune response in the colon of DSS‐induced rats after treatments. A) Representative IHC staining of TNF‐α in colon tissues of rats in each group after various treatments. B) Representative fluorescence staining of CD86 in colon tissues of rats in each group after treatments. C) Representative histological trichrome staining for collagen in colon tissues of rats in each group after treatments. D) Representative fluorescence staining of α‐SMA, CD31 in colon tissues of rats in each group after treatments. Scale bar = 100 µm.

Chronic colitis is associated with tissue inflammation. Histological analysis indicates typical features of colitis, including severe epithelial ulceration, loss of goblet cells, and neutrophil and lymphocyte infiltration in DSS‐treated rats. By contrast, animals treated with budesonide through the hydrogel have a more integrated glandular structure and less inflammatory cell infiltration (Figure S14A, Supporting Information). Immunohistochemistry and quantitative PCR (qPCR) demonstrate dramatically increased expression of pro‐inflammatory cytokine *TNF‐α* and infiltration of M1 macrophages (CD86^+^) in DSS‐treated colon, and budesonide treatment via endoscope‐guided delivery can significantly reduce the cytokine expression and inflammatory infiltration, compared with traditional drug delivery approach (**Figures** **6**A,B; Figures S14B,C, Supporting Information).

Ulcerative colitis can lead to tissue fibrosis, including abnormal collagen deposition and blood vessel formation at the lesion. Masson staining and qPCR analysis demonstrate diminished collagen expression and deposition when animals are treated with budesonide through hydrogel‐mediated delivery (Figure 6C; Figure S14D, Supporting Information). In line with these findings, compared with the conventional delivery route, endoscope‐guided drug delivery can more potently reduce the expression of *CD31* and *α‐SMA* in colon tissue, as determined by immunofluorescence and qPCR (Figure 6D; Figures S14E,F, Supporting Information). The treatment also reduces spleen enlargement (Figure S14G, Supporting Information) and intestinal bleeding. Blood analysis indicates elevated hemoglobin and red blood cells but decreased white blood cells in animals treated with endoscope‐guided delivery of budesonide (Figure S15, Supporting Information).

### 2.6 Hydrogel‐Mediated Drug Delivery Restores Intestinal Microbiota

The human intestinal tract contains ≥100 trillion microorganisms that play a pronounced function in regulating gut homeostasis by maintaining biological activities such as the mucosal barrier, and metabolic, and immune functions.^[^ ^17^ ^]^ Disturbances that occur in gut equilibrium will result in a reduction of beneficial bacteria and the growth of opportunistic pathogens and finally lead to various intestinal or systemic diseases.^[^ ^18^ ^]^

**Figure 7:**
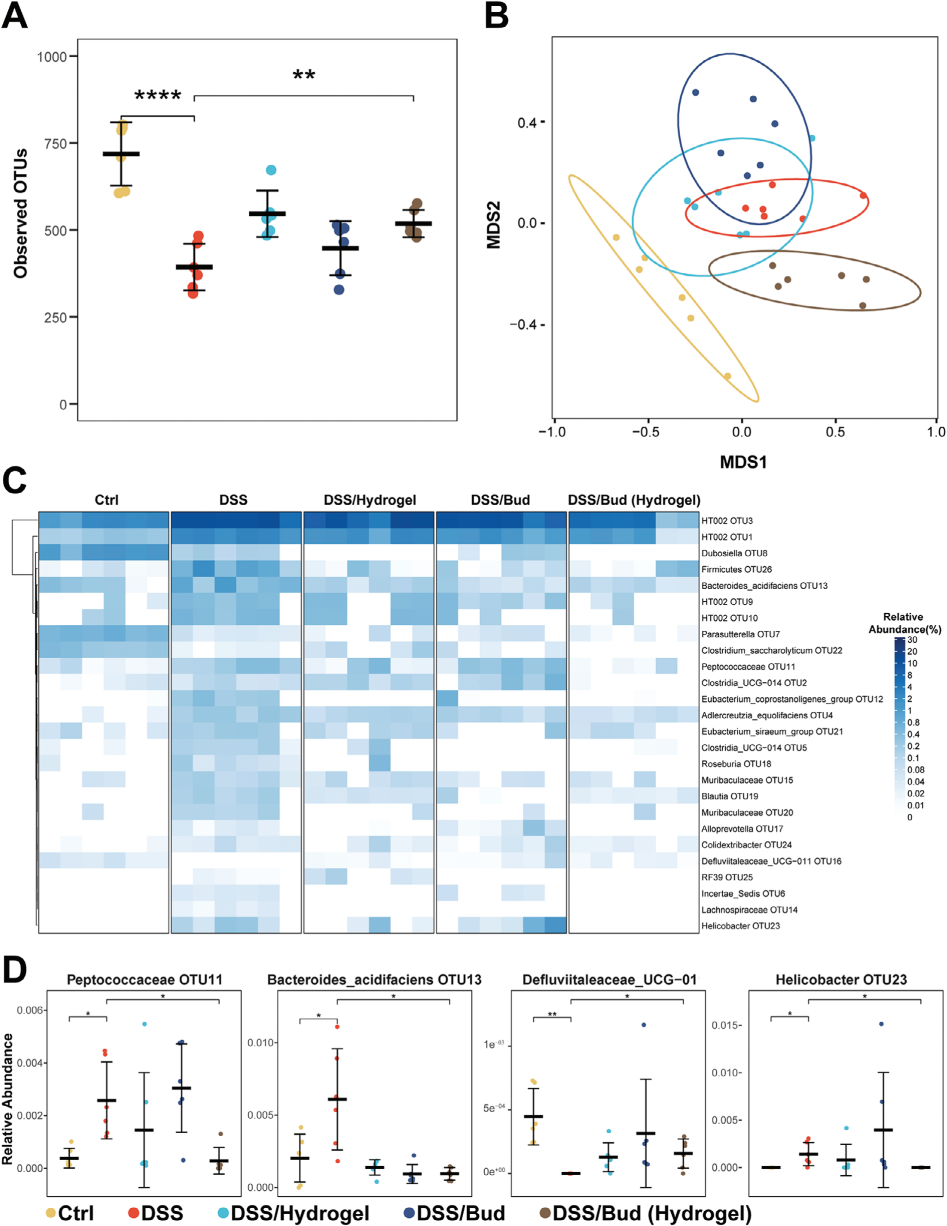
16S ribosomal RNA (rRNA) sequencing analysis of gut microbiota after therapy. A) Observed operational taxonomic units showed the α‐ diversity of the microbial community. B) Principal coordinates analysis showed the β‐diversity of the gut microbiome. The significance of clustering was determined using analysis of similarities (NMDS). C) Heatmap exhibited the relative abundance of microbial compositional profiling at a genus level. D) Relative abundance of select taxa. Data are presented as mean ± s.d. (*n* = 6). The unpaired two‐tailed Student's t‐test was used for comparison between the two groups. ^*^ *P* < 0.05, ^**^ *P* < 0.01, ^***^ *P* < 0.001, ^****^ *P* < 0.0001.

To investigate the potential role of hydrogel‐mediated drug delivery to intestinal microbiota, we collected the feces of rats and performed 16S rDNA sequencing of the samples to compare the diversity and conversion of gut flora in each group. As expected, DSS treatment of rats leads to a significant drop in taxon richness, indicating considerably decreased diversity and structure of the community. By contrast, HANB/HAMA hydrogel‐mediated delivery of budesonide significantly improved α – diversity, compared with conventional delivery as measured by observed operational taxonomic unit (OTU) (**Figure** **7**A). The non‐metric multidimensional scaling (NMDS) plots revealed distinct gut microbiota profiles for control or animals treated with budesonide with or without hydrogel‐mediated endoscopic delivery (Figure 7B and the total enterobacteria composition of each sample at the phylum and genus is shown in Figure S16, Supporting Information). Hydrogel‐mediated delivery of budesonide can significantly increase the abundance of Defluviitaleaceae_UCG‐011 (OTU16) and decrease the abundance of Bacteroides_acidifaciens (OTU13), Helicobacter (OTU23), and Peptococcaceae (OTU11) (Figures 7C,D). These results suggest that drug delivery via HANB/HAMA hydrogels can restore gut microbiota in the experimental colitis model, which can potentially suppress inflammation and promote the healing of lesions. Consistent with this notion, immunohistochemistry and qPCR analysis revealed enhanced expression of the anti‐inflammatory cytokine, *IL‐10* and elevated presence of M2 macrophages (CD206^+^) in colon tissues upon treatment with drug‐loaded hydrogel (Figure S17, Supporting Information).

## 3 Discussion

The incidence of IBD, including UC, is steadily increasing worldwide. More effective and safer treatments are urgently needed.^[^ ^19^ ^]^ Many patients with UC may benefit from topical therapy, as these diseases are limited to the distal colon/rectum. Hydrogel provides a new platform for colonic drug delivery, as hydrogel material can adhere to inflamed sites and protect the intestinal mucosa. However, traditional hydrogel materials are not suitable for UC treatment, due to low adhesiveness to mucosal membrane, poor colonic retention, rapid clearance, and significant swelling in vivo. In this study, we designed and tested a HANB/HAMA hydrogel for endoscope‐guided drug delivery, based on PTPC reaction‐based crosslinking to achieve spatiotemporal specificity for UC treatment at the lesion sites. In contrast to other injectable in situ forming hydrogels,^[^ ^20^ ^]^ the highly efficient PTPC reaction endows the HANB/HAMA hydrogel with physical robustness that withstands the in vivo dynamic environment. Moreover, the photo‐responsive generation of reactive aldehyde groups facilitates the spatiotemporally controlled adhesion in situ, differentiating the HANB/HAMA hydrogel from partially oxidized polysaccharides‐based hydrogel.^[^ ^6^, ^9^ ^]^ Our results demonstrate improved intestinal adhesion and retention, sustained drug release, and increased drug concentrations at sites of inflammation, as well as enhanced therapeutic efficacy for both budesonide and mesalazine when delivered with our platform.

Recent advances in endoscopic techniques have added new tools for better characterization and further treatment of intestinal disorders.^[^ ^21^ ^]^ While UC is continuous inflammation of the colon, CD is where healthy parts of the intestine are mixed in between inflamed areas and can occur anywhere along the digestive tract. Since endoscopes can be inserted deep into the human intestine, likely, our drug delivery platform may also benefit CD treatment. Similar to an endoscope, a laparoscope also has a camera and light source attached to the end of a long flexible tube that is inserted into the body cavity. In this regard, the optically responsive hydrogel system can be potentially applied via laparoscope, making it an attractive platform for abdominal surgery. For instance, gallbladder cancer is usually treated with laparoscopic surgery.^[^ ^22^ ^]^ Cancer treatment drugs can be loaded into our hydrogel system and applied laparoscopically to resection wounds to prevent tumor recurrence.

In conclusion, our endoscopic delivery strategy of mucosal‐adhesive drug‐loaded photo‐crosslinking hydrogels presents a strategy for the sustained and precisive treatment of UC, which will pave the way for future clinical translation of long‐acting hydrogels for the durable treatment of gastrointestinal diseases with minimally invasive medical interventions.

## 4 Experimental Section

Detailed experimental procedures are provided in the Supporting Information.

### Animal Ethics

The animal studies were carried out and approved by the Experimental Animal Center of Fudan University Shanghai Cancer Center (No. FUSCC‐IACUC‐S2024‐0710). Adult male Sprague‐Dawley rats (200–220 g) were employed to establish a model of chronic colitis. They were raised in specified pathogen‐free (SPF) animal barriers. Animals were housed in a room with a humidity of 55 ± 15% at a temperature of 22 ± 5 °C. Enough space for movement as well as plenty of food and drinking water were available in the cage.

### Statistical Analysis

Statistical analysis was conducted using GraphPad Prism v.9.0. The average of at least 3 replicates was taken to describe the entire population without assumptions on the statistical distribution. One‐way ANOVA followed by Tukey's post hoc test was used to assess the statistical significance (P value) of the difference for most experiments.

### Data Availability Statement

All relevant data and information can be found in the Article and its Supplementary Information. Additional primary data derived from this study are available on request from the corresponding authors. The source data are attached to this paper.

## Conflict of Interest

The authors declare no conflict of interest.

## Author Contributions

W.W., J.Z., X.Q., and T.C. contributed equally to this work. W.W., J.Z., L.Z., X.W., and Y.M. designed the experiments. W.W., X.Q., and T.C. performed the experiments and analyzed the data. J.L. performed the bioinformatics analysis. W.W., J.Z., and T.C. wrote the original draft. Y.M., L.Z, J.Z, and X.W. wrote the review & editing and supervised the project. All of the authors contributed to discussing the results and implications and editing the manuscript at all stages.

## Supporting information



Supporting Information

Supplemental Video 1

Supplemental Video 2

Supplemental Video 3

Supplemental Video 4

## Data Availability

The data that support the findings of this study are available from the corresponding author upon reasonable request.
