# Supplementary material for: Enabling Targeted Drug Delivery for Treatment of Ulcerative Colitis with Mucosal‐Adhesive Photoreactive Hydrogel
Source: Adv Sci (Weinh). 2025 Feb 3;12(12):2404836. doi: 10.1002/advs.202404836 (PMC11948015; doi:10.1002/advs.202404836)
Supplement: Supplementary file 1 — Supporting Information [file ADVS-12-2404836-s005.docx]

**Supporting Information**

**Enabling Targeted Drug Delivery for Treatment of Ulcerative Colitis with Mucosal-Adhesive Photoreactive Hydrogel**

Wen Wu, Jian Zhang, Xiao Qu, Ting Chen, Jinming Li, Yongzhi Yang, Lifeng Chen, Alex Hoover, Fanying Guo, Cheng Kong, Bingkun Bao, Qiuning Lin, Mengxin Zhou, Linyong Zhu^*^, Xiaoyang Wu^*^, Yanlei Ma^*^

**1. Experimental Section**

**The synthesis of HANB**

As shown in **Figure S2A,** **Supporting Information**, we described the synthesis of HANB in our previous report.^[^^1^^]^ HA (1 g, Mw of 340 kDa) and NB (37 mg, 0.1 mmol), which dissolved in 2 mL of dimethyl sulfoxide (DMSO) firstly, were dissolved in 50 mL of 2-morpholinoethanesulfonic acid solution (0.1 M, pH = 5.2). Then, 4-(4,6-dimethoxy-1,3,5-triazin-2-yl)-4-methyl morpholinium chloride (DMTMM, 0.2 g, 0.68 mmol) was added into the above solution and stirred for 12 h in the dark at 37℃. Then, the solution was dialyzed, lyophilized, and stored at -20℃. ^1^H nuclear magnetic resonance (NMR) analysis (**Figure S2C, Supporting Information**) and UV-vis spectra (**Figure S2G, Supporting Information**) were performed to calculate the substitution degree of the *o*-nitrobenzyl group.

**The synthesis of HAMA**

The synthesis route is shown in **Figure S2B, Supporting Information**. HA (4 g, Mw of 44 kDa) was dissolved in 100 mL DI water. Methacrylic anhydride (2.4 mL) was added into above HA solution at 4℃. Then, 5 M NaOH was added dropwise until the pH was maintained between 8 and 11. The reaction continued for 4 h in the dark at 4℃. The pH of above solution was adjusted to about 7 by 1 M HCl. The solution was centrifuged, and the supernatant was collected. Then, the supernatant was dialyzed and lyophilized. HAMA with different substitution degrees and molecular weight was also synthesized by same method except for the addition of methacrylic anhydride. ^1^H NMR analysis (**Figure S2D, Supporting Information**) was conducted to determine the substitution degree of the methacrylate as previously described.^[^^2^^]^

**The fabrication of HANB/HAMA hydrogels**

Hydrogel precursors of HANB (2, 4, 6%, w/v), HAMA (2.5, 5, 7.5‰, w/v), and LAP (1.2, 2.4, 3.6‰, w/v) were dissolved in PBS (pH 7.4). Then, the above precursor solutions were gelled under light irradiation (395 nm LED, 50 mW cm^-2^) for different measurements.

**Synthesis of drug-loaded hydrogel solution**

Mesalazine (Mes) was dissolved in PBS (pH 7.4) at a concentration of 50 mg mL^-1^. Then, hydrogel precursors of HANB (4%, w/v), HAMA (5‰, w/v), and LAP (2.4‰, w/v) were mixed in above solution at 37℃. For Budesonide (Bud)-loaded HANB/HAMA hydrogels, the Bud (0.5 mg) was firstly dissolved in 0.1 mL of dichloromethane (DCM), which was added dropwise into 1 mL of HANB/HAMA hydrogel precursors under vigorous vortex to form emulsion, which was transferred into an ice bath. Then the sonication (40% amplitude, 3 s on, 3 s off, 20 s) is required to further emulsify the above solution with a probe-type sonicator (Scientz Ultrasonic Homogenizer JY92-IIN). Subsequently, the organic phase was evaporated under mild vacuum condition to form the Bud-loaded HANB/HAMA hydrogel solution.

**Mechanical performance of HANB/HAMA hydrogels**

Mechanical tests were carried out by an INSTRON 68SC-1 testing machine with 100 N capacity. For tensile tests, hydrogel samples were prepared in a dumbbell-shaped molds (3.3×20×1.9 mm), and the samples were pulled to failure at a testing rate of 5 mm min^-1^. For compression tests, the hydrogel samples were prepared in cylindrical-shaped molds (10 mm diameter and 3 mm height). The cyclic compression tests were performed ten times for each strain at 10, 20, and 30% with speed set at 1mm min^-1^. The above hydrogels were completely gelled upon light irradiation (395 nm LED, 50 mW cm^-2^).

**Injectability of HANB/HAMA hydrogels**

A compression test was performed to measure the injection force of HANB/HAMA hydrogels through a flexible metal tube (0.6mm-outer diameter, 0.3 mm-inner diameter, and 500 mm-length). A 1 mL syringe containing HANB/HAMA hydrogels was placed and fixed on a universal testing machine (AI-3000, GOTECH, China). The injection force (force needed to maintain the continuous deployment of the hydrogels) were recorded. The experiment was performed three times to ensure data reliability.

**Rheological analysis**

Dynamic rheology experiments were carried out on a HAAKE MARS III photo-rheometer with parallel-plate (P20 TiL, 20 mm diameter) geometry and 395-nm LED (50 mW cm^-2^) at 37℃. Time sweep oscillatory tests were performed at a 10% strain [Controlled Deformation (CD) mode], 1-Hz frequency, and a 0.5-mm gap for 180 s. Strain sweeps were performed to verify the linear response. The gel point was determined as the time when the storage modulus (G′) surpassed the loss modulus (G″). The final storage modulus was determined and recorded as the final modulus of hydrogels.

**180-degree peel test**

To measure interfacial toughness, adhered samples with widths of 2.5 cm (W) were prepared and tested by the standard 180-degree peel test (ASTM F2256) using a mechanical testing machine (AI-3000, GOTECH). All tests were conducted with a constant peeling speed of 40 mm min^−1^. Poly (methyl methacrylate) films (with a thickness of 50 µm) were applied using cyanoacrylate glue (Krazy Glue) as a stiff backing for the hog casing. 100 μL of gel precursor was evenly covered on the surface of the hog casing. A second hog casing substrate was conducted as the former one and then placed on the first substrate to get the test sample. Then, test samples were irradiated with a 395-nm LED (50 mW cm^-2^), resulting in hydrogel gelling in situ. Two sides of test samples were fixed on the testing machine, and increasing pull strength was applied to one side. The measured force reached a plateau as the peeling process entered the steady state (F_plateau_). Then, the interfacial toughness of the hydrogel was calculated according to Eq. (1). Five samples were tested to determine the adhesive strength of each group (n = 5).

$Interaical toughness=\frac{2F_{plateau}}{W}$ (1)

**Lap shear tests of HANB/HAMA hydrogels**

The lap shear test was carried out as the previous method.^[^^3^^]^ Briefly, a 2.5 × 2.5 cm fresh hog casing was adhered tightly to one side of glass slide (8 × 2.5 cm) by commercial cyanoacrylate glue. Then, 100 μL of gel precursor was evenly covered on the surface of the hog casing. A second hog casing substrate was conducted as the former one and then placed on the first substrate to get the test sample. Then, test samples were irradiated with a 395-nm LED (50 mW cm^-2^), resulting in hydrogel gelling in situ. Two sides of test samples were fixed on the tensile testing machine (INSTRON 68SC-1), and increasing pull strength was applied to one side. Then, the tissue adhesive strength of the hydrogel was calculated according to Eq. (2). Five samples were tested to determine the adhesive strength of each group (n = 5).

$Shear strength=\frac{F_{max}}{A}$ (2), where F_max_ is the maximum pulling stress and A is the adhesive area.

***In vitro* swelling tests of HANB/HAMA hydrogels.**

The cylindrical hydrogel samples (10 mm diameter and 3 mm height) were produced and their initial weight were recorded (W_0_). Then, the above hydrogels were immersed totally in PBS (pH = 7.4) at 37℃ for complete swelling (n = 4). At different time points, these samples were gently collected and softly wiped with tissue paper to remove excess water. The mass was recorded as the swelling weight (W_t_) until the mass of hydrogels remained unchanged. The swelling ratio was calculated as the following Eq. (3).

$Swelling ratio=\frac{W_{t}}{W_{0}}\times100\%$	(3)

***In vitro* degradation of HANB/HAMA hydrogels**

The weight of above hydrogel samples after complete swelling was recorded as the initial weight W_0_. Then, hydrogels were immersed in PBS with addition of 20 U mL^-1^ hyaluronidase (from bovine testes, purchased from Sigma-Aldrich) at 37℃ (n = 4). The enzyme-containing solution was refreshed every day to ensure the enzymatic activity. At each time point, these samples were gently collected and wiped with tissue paper to discard surface liquid. The weight was recorded as W_t_, and the mass loss (%) was determined according to the following Eq. (4).

$Mass loss=\frac{W_{t}}{W_{0}}\times100\%$	(4)

***In vitro* drug release studies**

HANB/HAMA hydrogels (0.2 g) loaded with Budesonide (Bud) and Mesalazine (Mes) were immersed in PBS (1 mL, pH = 5.6, 7.4 or 9.0) under shaking (100 rpm) at 37℃, respectively. As shown in **Figure S9,** **Supporting Information**, the amount of Bud and Mes that released from the hydrogel were determined using UV/vis spectrometer and HPLC analysis. The HPLC apparatus was from the Waters e2695 separations module, equipped with a Waters 2489 UV/vis detector. HPLC was performed using the following specifications: mobile phase, 42% acetonitrile/ 58% water with 1‰ trifluoroacetic acid; column, XBridge C18 5 μm, 4.6×250 mm; flow rate, 1 mL min^-1^; injection volume, 10 μL; column temperature, 35 ℃; and the detect wavelength, 246 nm.

**Cell viability test of HANB/HAMA hydrogels**

NCM460 and Caco-2 cell lines were obtained from ATCC. The cell cytotoxicity of HANB/HAMA hydrogels was tested by coculture with NCM460, Caco - 2 cells in vitro. Cells were cultured in 24-well plates, and HANB/HAMA solution was added into the coculture system by a transwell chamber without impacting cell adhesion and proliferation. After gelation of HANB/HAMA hydrogels under light irradiation (395 nm LED, 50 mW cm^-2^), cell viability was assessed by CCK-8 assay and Calcein-AM/PI live/dead staining. GelMA was taken as the control group (Ctrl) for cell viability and wound-scratch assays.

**Colitis Induction and** **Drug Treatment Protocol**

Dextran Sodium Sulfate Induced Rat Colitis Model**:**^[^^4^^]^ After one week acclimation period, SD rats (n = 60) ad libitum drunk with 5 % DSS (DSS colitis grade, MP Biomedicals) water for one week, then replaced with regular water for one week, for a total of three cycles for inducing chronic enteritis, normal rats (n = 6) had free access to standard tap water. Endoscopic assessment at the end of 1, 3 and 5 weeks, to imitate the process of chronic ulcerative colitis, we randomly divided the DSS-induced model rats into different therapy groups after confirmed chronic colitis at the end of the first cycle intervention by body weight, endoscopy and DAI, therapy was started at the 3rd week. Healthy mice were fed only water as a control (n = 6, Ctrl), All colitis rats were randomly assigned into six groups, including DSS-induced chronic colitis rats treatment with PBS group (n = 10, DSS), DSS - induced chronic colitis rats treatment with HANB/HAMA group (n = 10, DSS/Hydrogel), DSS - induced chronic colitis rats treatment with Bud solution group ( n = 10, DSS/Bud), DSS - induced chronic colitis rats treatment with mesalazine solution group (n = 10, DSS/Mes), DSS - induced chronic colitis rats treatment with Bud loaded - hydrogel group (n = 10, DSS/Bud (hydrogel)), DSS - induced chronic colitis rats treatment with mesalazine loaded - hydrogel group (n = 10, DSS/Mes (hydrogel)). Weight changes were monitored daily throughout the experiment. For microbiome assays, rats were co-housed for 1 week until randomly assigned to each group to homogenize the intestinal microbiomes between individual rats, feces were collected on a scheduled day. Finally, the rats were sacrificed, blood samples were taken for cell counting, serum was extracted by centrifugation at 3000 rpm for 15 min for liver and kidney function analysis, and spleen weight and colon length were measured. Two 0.5 cm lengths of distal colon were then used for histological evaluation and immunofluorescence staining, and the remaining sections were used for qPCR assays.

Drug Treatment Protocol**:** Considering the sustained release effect of the drug-loaded hydrogel, to reduce the damage and irritation caused by endoscopic administration in rats, and to take into account patient compliance for subsequent clinical conversion, rats in DSS/Bud (hydrogel) and DSS/Mes (hydrogel) were administered every three days and rats in DSS/Bud and DSS/Mes once a day, Each treated group received an equal dose of mesalazine (40mg kg^-1^ d^-1^) and Bud(0.5 mg kg^-1^ d^-1^) either in the form of drug solution or drug-loaded HANB/HAMA hydrogels, with the dose of each drug converted per unit body weight kept consistent. DSS, DSS/Hydrogel accepted equal volumes of PBS and HANB/HAMA hydrogels, separately. We use the working channel of a small animal endoscope to introduce the hydrogel and photo-triggered it, as described previously.

**Fluorescence evaluation for HANB/HAMA hydrogels by IVIS Imaging**

ICG was incorporated in HANB/HAMA hydrogels as a fluorescence indicator to illustrate the adhesive ability of the HANB/HAMA hydrogels on the tissue surface according to a former study.^[^^5^^]^ ICG-labeled HANB/HAMA hydrogels were produced as the above method by replacing HANB with ICG-labeled HANB. ICG-labeled HANB were synthesized by condensation between ICG-Amine and carboxylic acid groups on HANB, while ICG-GelMA was synthesized by the same method. In order to generate an enema, the ICG - HANB/HAMA precursor were dissolved at a solid content of 1% (w/v).

Rats with DSS-induced colitis were fasted for 24 h, and their abdominal hair was shaved before imaging. The rats were given 1mL of ICG solution (Ctrl), ICG - GelMA, and ICG - HANB/HAMA by endoscopy, respectively. Fluorescence was detected using an IVIS Spectrum (AniView, China) at 0, 1 and 3 days after endoscopy. Representative colons of rats in each group with strong ICG - HANB/HAMA signal were removed and imaged with IVIS on days 0, 1, 2, and 3.

***In vivo* degradation of hydrogels.**

Male rats (~ 200 g) were conducted in the *in vivo* degradation assays. A 1-centimeter incision was made either on the dorsal inner skin or abdominal area of the rats. Hydrogel samples (n = 5; 10 × 2 mm cylinders) were implanted under sterile conditions. The rats were sacrificed after 6 weeks for biodegradation studies, which included measuring the diameter and calculating the degradation rate of hydrogels. The degradation rate (%) was determined by dividing the mass loss of the hydrogels by its initial mass at the time of implantation.

***In vivo* pharmacokinetic study**

According to the above-described protocols, after DSS-induced ulcerative colitis in rats was formed, rats were administered equal doses of drugs by gavage or endoscopic, respectively. Bud-loaded HANB/HAMA hydrogels (1.5 mg kg^-1^) (Bud (hydrogel) - E group) were delivered and inspired by the endoscopy, matching drug solutions were administered by gavage (Bud - G group) or enema (Bud - E group). blood samples were acquired at time points (0, 0.25, 0.5, 1, 2, 4, 6, 8, 12, 24 and 48 h) and colon tissues were harvested at time points (0, 0.5, 1, 2, 4, 8, 12, 24 and 48 h) in Bud - G group, Bud - E group and Bud (hydrogel) - E group. The whole blood samples were instantly centrifuged at 3500 rpm for 10 min to get plasma. All collected plasma and colon tissue were stored at - 80℃.

**Quantitation of Bud by UPLC-MS/MS.**

Bud was quantitated in rat plasma and colon by UPLC-MS/MS (Agilent G6460A triple quadrupole mass spectrometer with an electrospray ionization source (ESI), Agilent 1200 pump, and auto-sampler, SB-C18 column (Bud, 2.1×75 mm, 2.7μm). All agents used for sample preparation and UPLC-MS/MS analyses are LC-MS grade (Merck). In this experiment, acetonitrile (ACN) and aqueous solution (containing 5 mM ammonium acetate) were used to separate Bud in the isocratic elution procedure (55: 45, V: V). The elution process involved a gradient of 95% to 20% of phase A from 0 to 2 minutes, and then a constant 20% of phase A was used from 2 to 4 minutes. An ESI of negative ions adopted multi-reaction monitoring (MRM) mode, which was used for mass spectrometry. Sheath flow gas and drying gas were both nitrogen, the former was set at 320℃ and transported at a speed of 10 L min^-1^, while the latter was at 400℃ and 12 L min^-1^. High-purity nitrogen was used as collision gas at a pressure of 45 psi. The capillary voltage was kept at 4500 V. **Table S3** lists the parameters of Bud and IS in mass spectrometry detection. Biological samples were pre-treated via protein precipitation. For the colon, each tissue was weighed(mg) and added with PBS(μL) in a mass/volume of 1:3, homogenized, centrifuged, and then the supernatant was preserved for later use. 50 μL of the sample (plasma or the supernatants of colon tissue) was added into 150μL acetonitrile spiked with internal standard. Samples were vortexed for 2 minutes and centrifuged at 14,300 rpm for 10 minutes at 4℃, and 10 µL of supernatant was used for UPLC-MS/MS analysis. Standards were prepared similarly using blank plasma or tissue homogenates with 10 μL of spiking solution. Retention times were determined for each drug using The MassHunter Qualitative Analysis Software. All quantifications were calculated using the ratio of analyte peak area to internal standard peak area. The drug concentration acquired from the tissue supernatant by LC-MS was finally converted to quantity per unit weight of tissue. Calculations were made for key pharmacokinetic parameters such as maximum colonic/plasma concentration (Cmax), time to reach Cmax (Tmax), area under the colonic/plasma drug concentration-time curve (AUC), and DDI (Drug Delivery Index). The DDI was calculated according to the following: DDI^[^^6^^]^ = (AUC _bud (hydrogel)-E_ of drug in colon / AUC _Bud-E_ of drug in colon) / (AUC _bud (hydrogel)-E_ of drug in plasma / AUC _Bud-E_ of drug in plasma).

**Macroscopic Assessment of Colitis**

To assess the effect of treatment in different groups, the DAI of the rats was calculated at the end of each week according to changes in body weight, fecal status and rectal bleeding. Briefly, weight change was scored on grades 0 to 4: no weight loss, 0; 1-5% weight loss, 1; 5-10% weight loss, 2; 10- 20% weight loss, 3; and > 20% weight loss, 4. In terms of fecal consistency, a score of 0 for formed stools, 2 for pasty and 4 for liquid. Bleeding was graded as 0 for no blood, 2 for less bleeding, and 4 for severe bleeding. The sum of those scores is the DAI, which ranges from 0 (healthy) to 12 (most severe colitis).

**Endoscopic Analysis of Colitis**

The severity of colitis in rats was assessed by using the small animal endoscopic system described above. Brieﬂy, at the end of the study, rats were anesthetized with isoﬂurane, and the colon was examined endoscopically after air inhalation. In order to quantify the severity of colitis, we used MES^[^^7^^]^ to assess and two doctors (Q.X. and W.W.) checked the endoscopic image once more and adjudicated the scores. The colitis is classified into grades 0-3 as follows: 0, normal or asymptomatic; 1, mild lesion with erythema, reduced vascular pattern, and mild friability; 2, moderate lesion with marked erythema, absence of vascular pattern, friability, and erosion; and 3, severe lesion with spontaneous bleeding and ulceration.

**Histological Evaluation**

1 cm distal colon slices were first fixed in 4% (v/v) formalin and 70% (v/v) ethanol followed by paraffin embedding, and deparaffinized were stained with hematoxylin-eosin (HE) and Masson's trichrome. As previously described,^[^^8^^]^ the severity of histological damage to the colon was scored in a blinded fashion to avoid observer bias. Briefly, colonic damage was graded as follows: 0, normal; 1, hyperplasia, irregular crypts, and goblet cell loss; 2, mild to moderate crypt loss (10 - 50%); 3, severe crypt loss (50 - 90%); 4, complete crypt loss with intact surface epithelium; 5, small to medium-sized ulcers (crypts <10mm wide) and 6, large ulcers (crypts >10mm wide). Inflammatory cell infiltration was rated respectively for the mucosa (0, normal; 1, mild; 2, modest; 3, severe), submucosa (0, normal; 1, mild to modest; 2, severe) and muscle/serosa (0, normal; 1, moderate to severe). The scores of epithelial damage and inflammatory cell infiltration were added to give a total score from 0 to 12.

**Immunofluorescence**

For immunofluorescence staining, the deparaffinized sections were undergone epitope retrieved and blocked by incubating with BSA for 45 minutes at room temperature. The sections were then exposed to diluted primary antibodies and incubated at room temperature for 1 hour. After washing with PBS, the sections were incubated with secondary antibodies at room temperature for 1 hour and then fixed with DAPI-containing mounting medium. The primary antibodies used were rabbit monoclonal CD86 (13395-1-AP, Proteintech®), CD206 (ab64693, Abcam®), CD31 (ab222783, Abcam®) and α-SMA (ab7817, Abcam®). TUNEL staining was performed according to Sigma-Aldrich instructions. The sections were subjected to confocal laser scanning microscopy (Nikon, Ti-E&A1plus) after staining with donkey anti-rabbit IgG Alexa Fluor 488 secondary antibody (ab150073, 1:1000, Abcam®).

**Immunohistochemistry**

For immunohistochemistry analysis, after deparaffinization, sections were blocked with PBS containing 5% bovine serum albumin (BSA) and 0.1% Tween 20 (PBST), diluted primary antibodies were added and incubated overnight at 4℃. Subsequently, the sections were washed with PBST and incubated with secondary antibodies at 37℃. The main primary antibodies were rabbit monoclonal TNF-α (ab307164, 1:100, Abcam®) and mouse monoclonal IL-10 (60269-1-Ig, 1:200, Proteintech®). The secondary antibodies were goat anti-rabbit IgG H&L (HRP) or goat anti-mouse IgG H&L (HRP). The bound antibodies were detected using the DAB chromogenic kit (8801-4965-72, eBioscience™) after extensive washing.

**Quantitative reverse transcription PCR**.

According to the manufacturer's instructions, colon RNA was extracted using Trizol reagent (Invitrogen, USA). Subsequently, cDNA reverse transcription was conducted with a kit from Thermo Fisher Scientific. Real-time PCR was thenrun with BeyoFast™ SYBR Green One-Step qRT-PCR Kit. To calculate the relative mRNA fold changes, the 2−ΔΔCt method was utilized. The primers utilized in the study are featured in **Table S4**.

**Microbiome (16S) Sequencing and Analysis**

Feces samples were finally sampled for 16S rDNA sequencing analysis. In summary, the isolated DNA was utilized to utilize 16S rDNA libraries for the NovaSeq platform sequencing analysis. Alpha diversity was evaluated for Chao1, observed species, Shannon and Simpson indices. Species displaying significant differences were investigated using the t-test. The cladogram showed a taxonomic tree of difference-rich taxa by performing LEfSe analysis on genera.

***In Vivo* Toxicity Analysis**

Rats were euthanized and their main organs, namely the heart, liver, lungs, kidneys, and spleen, were preserved for further analysis by fixing them in 4% (v/v) paraformaldehyde and 70% (v/v) alcohol, and embedded in paraffin. Tissues sectioned were analyzed for biochemical assessment after staining with H&E via microscopy. Blood samples were taken, and the serum was acquired through centrifugation at 1000 g for 10 minutes. The blood indices comprised the white blood cell (WBC) count, red blood cell (RBC) count, and hemoglobin (HGB) levels. The serum biochemistry measurements, comprising alanine aminotransferase (ALT), aspartate aminotransferase (AST), alkaline phosphatase (ALP), creatinine (CREA), blood urea nitrogen (UREA), and Uric Acid (UA) were analyzed through an automated chemistry analyzer (Nihon Kohden Co.).

**2. Figures/video legends**

**Figure S1. Gelation and integration mechanism of HANB/HAMA hydrogel for the endoscopy delivery for treatment of chronic colitis rats. (A)** Schematic illustration of mechanisms for HANB/HAMA hydrogels construction. **(B)** The combination of endoscopic injectability and photo-crosslinking makes the HANB/HAMA hydrogels colon mucosal repair system exceedingly convenient. **(C)** Spraying HANB/HAMA hydrogels to cover the inflamed area promotes healing by integrating with the host colon tissue, prevents pathogen invasion, and prolongs colon retention for sustained precision therapy.

**Figure S2. The synthesis and characterization of HANB and HAMA. (A-B)** The synthetic route of **(A)** HANB and **(B)** HAMA. **(C-F)** The ^1^H NMR spectra of **(C)** HANB and **(D-F)** HAMA. **(G)** The linear relationship between absorbance at 345 nm wavelength and concentration of NB.

**Figure S3. Compression tests of HANB/HAMA hydrogels.** **(A)** Representative compressive stress-strain curves of HANB/HAMA hydrogels with solid contents of 2.37, 4.74, and 7.11%, respectively. **(B)** Successive loading–unloading cycling compressive tests of the HANB/HAMA hydrogels (4.74%), conducted five times at each strain ratio: 10%, 20%, and 30%.

**Figure S4. Rheology analysis of hydrogels.** **(A)** Rheological time sweeping of the HAMA-*4* hydrogel and the HANB/HAMA hydrogels with solid contents of 2.37, 4.74, and 7.11%. The gray rectangle indicates the test without irradiation. The gelation points were defined as the time that G’’ surpass G’. **(B)** Rheological stress sweeping of the HANB/HAMA hydrogels with solid contents of 2.37, 4.74, and 7.11% before and after irradiation.

**Figure S5. The stability and biodegradability of HANB/HAMA hydrogels in vitro.** **(A)** The representative visual changes of the HANB/HAMA hydrogels in PBS with various pH (pH=5.6, 7.4, and 9.0). **(B)** The swelling ratio of the HANB/HAMA hydrogels in PBS with various pH (pH=5.6, 7.4, and 9.0). **(C)** In vitro degradation profile of HANB/HAMA hydrogels (solid content of 4.74%) with hyaluronidase (20, 50, and 100 U ml^-1^). Data are presented as mean ± s.d. (*n* = 3).

**Figure S6. Cytotoxicity experiments of HANB/HAMA hydrogels. (A)** NCM460 and Caco-2 cells were incubated with the HANB/HAMA, and cell viability was assessed with CCK-8 assays and Live/Dead staining. **(B-C)** Detection of CCK-8 to quantify cell viability in NCM460 cells **(B)** and Caco-2 cells **(C)** culture at Day 1 and Day 3 in control, GelMA and HANB/HAMA groups. **(D-E)** Live/Dead staining of NCM460 cells **(D)** and Caco-2 cells **(E)** loaded GelMA and HANB/HAMA on Day 1 and Day 3. Data are presented as mean ± s.d. (n = 3). Statistical analysis was performed using two-way ANOVA with Tukey’s multiple comparison test. ns P > 0.05, each group versus Ctrl.

**Figure S7. *In vivo* degradation assay. (A)** Macroscopic appearance of hydrogels implanted subcutaneously and intraperitoneally in rats after 42 days post-implantation. Black and red arrows indicate GelMA and HANB/HAMA hydrogels, respectively. Scale bar = 1cm. **(B)** The diameter of the implanted hydrogel corresponds to A. **(C)** The *in vivo* degradation rate of hydrogels after 42 days post-implantation based on weight measurements. Data are presented as mean ± s.d. (n = 5). Statistical analysis was performed using an unpaired two-tailed Student t-test. ns P > 0.05, *P < 0.05.

**Figure S8. Hydrogels adhesion test for endoscopy.**

**Figure S9. The quantification of drug release from drug-loaded HANB/HAMA hydrogels. (A-B)** The cumulative release of Budesonide **(A)** and Mesalazine **(B)** from the Bud and Mes-loaded HANB/HAMA hydrogels, respectively. Data are presented as mean ± s.d. (*n* = 10).

**Figure S10. Bud-loaded HANB/HAMA hydrogel exerts stronger efficacy in chronic colitis rats. (A)** The representative colonoscopy images of each group at the endpoint. **(B)** Mayo endoscopic scores change in each group at the end of 1, 3, 5, and 6 weeks. Data are presented as mean ± s.d. (n = 10). Statistical analysis was performed using one-way or two-way ANOVA with Tukey’s multiple comparison test. *P < 0.05, ** P < 0.01, *** P < 0.001, **** P < 0.0001, DSS/Bud (hydrogel) versus DSS.

**Figure S11. Mes-loaded HANB/HAMA hydrogel also displayed better efficacy in chronic colitis rats. (A-B)** Week bodyweight **(A)** and disease activity index (DAI) changes **(B)** in each group for 6 weeks. **(C)** Quantitative colon length of each group at the end of the experiment. **(D)** Colonic damage scores of each group at the end of the experiment. Data are presented as the mean ± s.e.m. (n=10). Statistical analysis was performed using two-way or one-way ANOVA with Tukey’s multiple comparison test. *P < 0.05, **P < 0.01, ***P < 0.001, ****P < 0.0001, DSS/Mes (hydrogel) or DSS/Mes versus DSS. # P < 0.05, ## P < 0.01, ### P <0.001, #### P <0.001, DSS/Mes (hydrogel) versus DSS/Mes.

**Figure S12. Liver and kidney function tests.** **(A-C)** The levels of alanine transaminase (ALT) (**A)**, aspartate aminotransferase (AST) (**B**), and alkaline phosphatase (ALP) (**C**) of mice in each group after therapy. (**D-F)** The level of blood urea nitrogen (UREA) (**D**), Uric Acid (UA) (**E**), and creatinine (CREA) (**F**) of mice in each group after therapy. Data are presented as the mean ± s.d. (n=5). Statistical analysis was performed using one-way ANOVA with Tukey’s multiple comparison test. ns P > 0.05, each therapy group versus DSS.

**Figure S13. Biocompatibility Test.** HE staining of major organs. Scale bar = 200 µm.

**Figure S14. Bud-loaded HANB/HAMA hydrogels inhibited inflammation and alleviated fibrosis. (A)** Representative histological sections of colons by H&E staining. **(B-F)** RT-PCR of *Tnfa* **(B)**, *Cd86* **(C)**, *Col1a1* **(D)**, *Acta2* **(E)**, and *Pecam1* **(F)** in colon tissues of rats in each group after treatments. **(G)** The spleen weight ratio of mice in each group. Data are presented as the mean ± s.d. (n=6). Statistical analysis was performed using one-way or two-way ANOVA with Tukey’s multiple comparison test. *P < 0.05, ** P < 0.01, *** P < 0.001, **** P < 0.0001, DSS/Bud (hydrogel) versus DSS.

**Figure S15. Improved hematological indicators related to anemia after treatment in each group. (A-C)** Hematology data of hemoglobin (HGB) **(A),** red blood cell (RBC) **(B)**, and white blood cell (WBC) **(C)** of rats in each group. Data are presented as the mean ± s.d. (n=10). Statistical analysis was performed using one-way ANOVA with Tukey’s multiple comparison test. *P < 0.05, **P < 0.01, *** P < 0.001, **** P < 0.0001, DSS/Bud (hydrogel) or DSS/Bud versus DSS.

**Figure S16. The general enterobacteria composition of each sample at the phylum and genus level.** (**A-B)** The community histogram showed the microbial compositional profiling at the phylum (**A**) and genus (**B**) levels. Data are presented as mean ± s.d. (n =6).

**Figure S17. Enhanced anti-inflammatory response in colon tissue after treatment. (A)** Representative IHC staining of anti-inflammatory cytokines IL-10 in colon tissues of rats in each group after various treatments. **(B)** Representative fluorescence staining of CD206 in colon tissues of rats in each group. **(C-D)** RT-PCR of *Il-10* **(C)** and *Mrc1* **(D)** in colon tissues of rats in each group after treatments. Data are presented as the mean ± s.d. (n=6). Statistical analysis was performed using one-way ANOVA with Tukey’s multiple comparison test. *P < 0.05, ** P < 0.01, *** P < 0.001, **** P < 0.0001, DSS/Bud (hydrogel) or DSS/Bud versus DSS.

**Video S1. Process of hydrogel photo-gelation in vitro**

The HANB/HAMA hydrogels can gel rapidly in situ within 4 s under light irradiation (395 nm LED, 50 mW cm^-2^).

**Video S2. Flushing HANB/HAMA hydrogel formed in situ on tissue surface.**

**Video S3. Photoexcited firmly adheres to inflamed colonic mucosa.**

The video illustrates the application of HANB/HAMA hydrogel precursor to the inflamed colon mucosa of rats, followed by transient irradiation with 395 nm UV light, which rapidly triggers the gelation of HANB/HAMA and adherence to the lesioned mucosa, even after further shaking or bending the colon, the gel remains firmly anchored to the mucosa and stable.

**Video S4. Endoscopic HANB/HAMA injection and photoexcitation process.**

Demonstration of the process of pressurized injection of HANB/HAMA hydrogels through the endoscopic channel and photoreactive of the hydrogels to adhere to the mucosa.

**References:**

[1]	Y. Yang, J. Zhang, Z. Liu, Q. Lin, X. Liu, C. Bao, Y. Wang, L. Zhu, *Adv Mater* **2016**, *28* (14), 2724.

[2]	S. K. Seidlits, Z. Z. Khaing, R. R. Petersen, J. D. Nickels, J. E. Vanscoy, J. B. Shear, C. E. Schmidt, *Biomaterials* **2010**, *31* (14), 3930.

[3]	Y. Yang, J. Zhang, Z. Liu, Q. Lin, X. Liu, C. Bao, Y. Wang, L. Zhu, *Advanced Materials* **2016**, *28* (14), 2724.

[4]	T. Manon-Jensen, S. Sun, M. Lindholm, V. Domislović, P. Giuffrida, M. Brinar, G. Mazza, M. Pinzani, Z. Krznarić, A. Di Sabatino, M. A. Karsdal, J. H. Mortensen, *United European Gastroenterol J* **2021**, *9* (2), 268.

[5]	Q. Mao, H. Pan, Y. Zhang, Y. Zhang, Q. Zhu, Y. Hong, Z. Huang, Y. Li, X. Feng, Y. Fang, W. Chen, P. Chen, B. Shen, H. Ouyang, Y. Liang, *Bioact Mater* **2023**, *19*, 251.

[6]	R. N. Fedorak, B. Haeberlin, L. R. Empey, N. Cui, H. Nolen, 3rd, L. D. Jewell, D. R. Friend, *Gastroenterology* **1995**, *108* (6), 1688.

[7]	K. W. Schroeder, W. J. Tremaine, D. M. Ilstrup, *N Engl J Med* **1987**, *317* (26), 1625.

[8]	Y. Lee, K. Sugihara, M. G. Gillilland, 3rd, S. Jon, N. Kamada, J. J. Moon, *Nat Mater* **2020**, *19* (1), 118.
